# Burden of obesity in patients undergoing dialysis, and hopes associated with semaglutide treatment for transplant listing: An interview study

**DOI:** 10.1111/dom.70011

**Published:** 2025-08-25

**Authors:** Lenka Vanek, Elias Stix, Amelie Kurnikowski, Simon Krenn, Sebastian Mussnig, Janosch Niknam, Manfred Hecking

**Affiliations:** ^1^ Department of Medicine III, Division for Nephrology and Dialysis Medical University of Vienna Vienna Austria; ^2^ Center for Public Health, Department of Epidemiology Medical University of Vienna Vienna Austria; ^3^ Clinic Landstraße, 1st Department of Medicine Vienna Austria; ^4^ KfH Kuratorium für Dialyse und Nierentransplantation e.V. Weiden Bavaria Germany

**Keywords:** chronic kidney disease, dialysis, gender, kidney failure, nephrology, obesity, semaglutide

## 1 BACKGROUND

Chronic kidney disease (CKD) and obesity are major global health challenges, with an increasing overlap in patients experiencing both kidney failure and high body mass index (BMI).^1^ Obesity has been shown to elevate the risk of complications of kidney transplantation, prompting the introduction of BMI thresholds for kidney transplantation listing.^1^, ^2^ However, this approach is controversial, as evidence suggests that timely transplantation benefits patients regardless of their weight^3^ and most patients with kidney failure are unable to meet these BMI requirements using conventional obesity management strategies.^2^

Although existing research has explored the experiences of patients undergoing dialysis and kidney transplantation,^4^, ^5^, ^6^ there is limited evidence on how obesity affects the health of patients with kidney failure.^7^, ^8^, ^9^ This study aimed to capture the experiences of patients with obesity undergoing dialysis, identify the barriers they face in treatment, and explore their expectations for obesity management to improve their overall health and eligibility for kidney transplant listing.

## 2 METHODS

In reporting this study, we followed the Consolidated Criteria for Reporting Qualitative Health Research. We recruited adult patients undergoing dialysis who had a BMI in excess of 30 kg/m^2^, and represented diverse backgrounds in terms of age, gender, dialysis vintage and transplantation history. The study was carried out between July and December 2023 at the Kuratorium for Dialysis and Transplantation e.V. Kidney Center Weiden (Germany), and the General Hospital Vienna (Austria). The study was approved by the Ethics Committee of the Medical University of Vienna (Nr. EK1363/2016).

Informed by existing literature and team discussions, we developed a semi‐structured interview guide focusing on the impact of obesity on CKD, dialysis and transplantation (Supplemental Methods 1 & 2). All interviews were conducted via telephone by one interviewer (LV), audio‐recorded and transcribed verbatim, with transcripts made available to the participants upon request.

We applied the principles of grounded theory to analyse the transcripts. Data saturation determined the sample size, ending collection when no new insights emerged. Initial concepts were developed into themes and subthemes through investigator triangulation by authors LV, ES and MH.

## 3 RESULTS

A total of 25 patients took part in the interviews. Most were men (56%), all White, with a mean BMI of 35.52 kg/m^2^. Most were in their 50s (28%) or 60s (36%), married (56%) and unemployed (55%, excluding retirees). Reported causes of kidney disease included diabetes (28%), hypertension (20%) and IgA‐nephropathy (20%). Mean dialysis vintage was 3.75 years, and 84% of patients had never received a kidney transplant. Demographic data and self‐reported clinical characteristics are included in Table S2.

**FIGURE 1:**
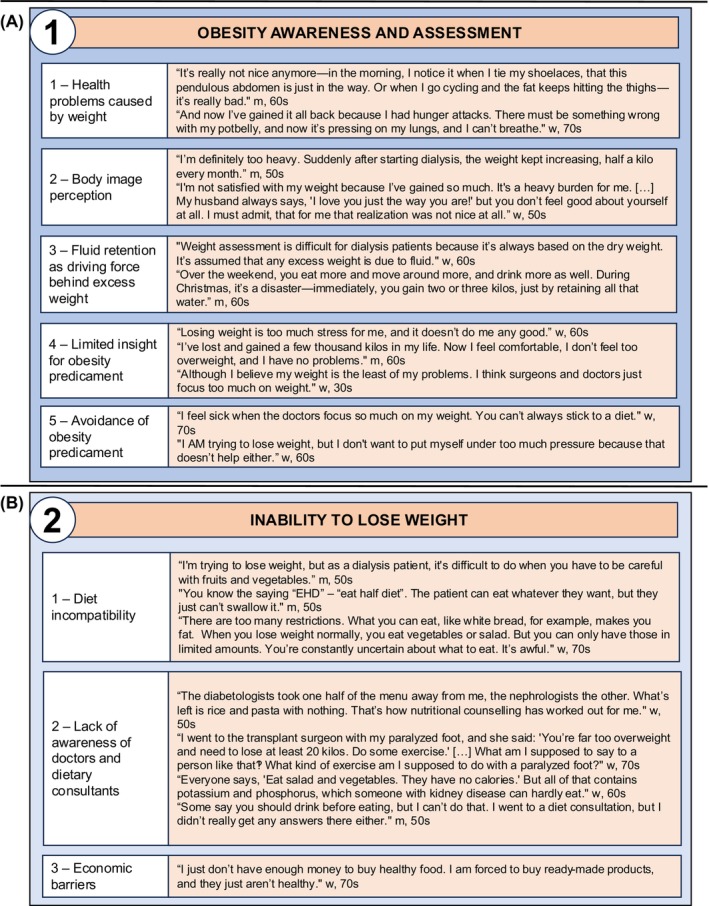
Experiences of patients with obesity undergoing dialysis. (A) Theme 1 with subthemes and supporting quotes. (B) Theme 2 with subthemes and supporting quotes. m, man patient; w, woman patient.

**FIGURE 2:**
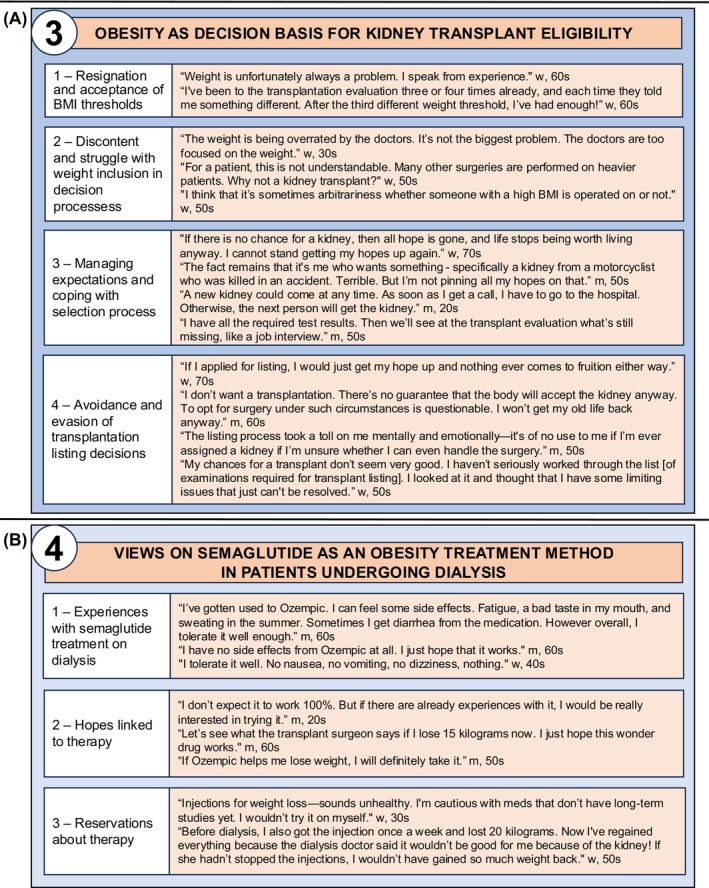
Experiences of patients with obesity undergoing dialysis. (A) Theme 3 with subthemes and supporting quotes. (B) Theme 4 with subthemes and supporting quotes. m, man patient; w, woman patient.

We identified four key themes related to the burden of obesity, which are listed with supporting quotes in Figures 1 and 2. The Supplement includes three additional aspects beyond the scope of obesity.

### 3.1 Theme 1—Obesity awareness and assessment

Weight and obesity assessment in patients undergoing dialysis elicited conflicting reactions, grouped into five subthemes (Figure 1A). Subtheme 1—health problems caused by weight‐– reflects patients' views linking their obesity to declining health and overall deterioration. In subtheme 2—body image perception—patients described how obesity negatively impacted their mental health and self‐image, not just their physical well‐being. Weight assessment in patients undergoing dialysis was not always straightforward, with quotes related to fluid retention as a driving force behind excess weight yielding subtheme 3. Subtheme 4—limited insight into their obesity status‐– emerged from participants showing little to no concern about their obesity, while subtheme 5—avoidance of obesity predicament—captured patients who deflected and minimized the issue, downplaying obesity's significance in their overall health.

### 3.2 Theme 2—Inability to lose weight

Patients reported having tried a variety of methods for weight reduction; however, they agreed that these measures were only applicable to a limited degree to those undergoing dialysis and voiced that they struggled with commonly practised methods for treatment of obesity. We divided the described struggles into three subthemes (Figure 1B). Subtheme 1—diet incompatibility—reflects the mismatch between common weight loss diets and the dietary restrictions of kidney patients. Subtheme 2—lack of awareness among doctors and dietary consultants—highlights patients' frustration with medical professionals often overlooking the limitations imposed by kidney failure and dialysis. In subtheme 3, we identified economic barriers to weight loss as a concern for some patients.

### 3.3 Theme 3—Obesity as a decision basis for kidney transplantation eligibility

By examining patients' differing views on how obesity affected their eligibility for transplant, we identified four subthemes (Figure 2A). Subtheme 1—resignation and acceptance of BMI thresholds—including patients who acknowledged or accepted the rationale behind BMI‐based transplantation criteria. Subtheme 2—discontent and struggle with weight inclusion in decision processes—captured the frustration of those who did not understand or agree with being excluded from transplantation due to obesity. Subtheme 3—managing expectations and coping with the selection process—reflected the emotional toll of prolonged dialysis, treatment pressure and transplant denial. In subtheme 4—avoidance and evasion of transplantation listing—we identified reasons why patients bypassed or shunned transplantation altogether.

### 3.4 Theme 4—Views on semaglutide as a method for treatment of obesity in patients undergoing dialysis

We identified three subthemes regarding patient perspectives on semaglutide treatment (Figure 2B). Subtheme 1—previous experiences with semaglutide treatment on dialysis—includes accounts from six patients who were receiving the therapy at the time of the interviews, all reporting mild‐to‐no adverse effects. Subtheme 2—hopes linked to therapy—reflects patients' interest in using semaglutide to support weight loss and, potentially, to meet the BMI threshold for transplantation. Subtheme 3—reservations regarding therapy—captures both patient concerns about starting the treatment and the caution previously expressed by their physicians about its continued use during dialysis.

## 4 DISCUSSION

In this study, we identified four key themes related to the burden of obesity. We observed major differences in the subjective perception of the relevance of obesity for one's ill‐health and in the agreement with medical diagnoses of overweight. Many felt reduced to their weight in healthcare interactions, reported inconsistencies in transplant listing decisions, and called for greater transparency. Concerns about being denied care based solely on obesity without a full health assessment were prevalent. This aligns with previous findings of weight stigma and BMI overemphasis,^10^ and of perceived injustice of transplantation rejections based on age or comorbidities.^6^

Although guidelines by Kidney Disease: Improving Global Outcomes (KDIGO) recommend supporting weight loss rather than excluding patients from transplant listing due to obesity, BMI thresholds are still implemented to prevent complications.^11^, ^12^ Patients undergoing dialysis often struggle to meet these targets,^2^, ^9^ find weight expectations unrealistic and consider popular diets unsuitable in kidney disease^11^ citing restrictive regimen, reduced mobility, treatment side effects and lack of interest in weight management centres, weight‐loss surgery, or medication‐based interventions^8^ as major barriers. Our patients described repeated, unsuccessful weight‐loss attempts and perceived a lack of provider expertise in effective strategies tailored to kidney failure. This study included six participants from an observational study on semaglutide for weight loss in dialysis patients, showing a mean BMI reduction of 1.5 kg/m^2^ (4.6 kg) and several successful listings.^13^ Together with the positive, first‐hand patient experiences reported here, our findings highlight a promising treatment avenue. This study's strength lies in its patient‐centred approach, highlighting practical barriers, the need for clearer dietary guidance, and promising early experiences with semaglutide. While individual experiences and regional specificity may limit generalizability, data validity was supported through member checking and investigator triangulation.

## CONFLICT OF INTEREST STATEMENT

The authors declare no conflicts of interest.

## PEER REVIEW

The peer review history for this article is available at https://www.webofscience.com/api/gateway/wos/peer‐review/10.1111/dom.70011.

## Supporting information


**Data S1.** Supporting information.

## Data Availability

All data generated or analyzed during this study are included in this article and its supplementary material files. Further inquiries can be directed to the corresponding author.

## References

[1] Friedman AN , Kaplan LM , le Roux CW , Schauer PR . Management of obesity in adults with CKD. J Am Soc Nephrol. 2021;32:777‐790. doi:10.1681/ASN.2020101472 33602674 PMC8017542

[2] Chintam K , Chang AR . Strategies to treat obesity in patients with CKD. Am J Kidney Dis. 2021;77:427‐439. doi:10.1053/j.ajkd.2020.08.016 33075388 PMC7904606

[3] Krishnan N , Higgins R , Short A , et al. Kidney transplantation significantly improves patient and graft survival irrespective of BMI: a cohort study. Am J Transplant. 2015;15:2378‐2386. doi:10.1111/ajt.13363 26147285

[4] Tommel J , Evers AWM , van Hamersvelt HW , et al. “What matters to you?”: the relevance of patient priorities in dialysis care for assessment and clinical practice. Semin Dial. 2023;36:131‐141. doi:10.1111/sdi.13080 35388533

[5] Palmer SC , Hanson CS , Craig JC , et al. Dietary and fluid restrictions in CKD: a thematic synthesis of patient views from qualitative studies. Am J Kidney Dis. 2015;65:559‐573. doi:10.1053/j.ajkd.2014.09.012 25453993

[6] Calestani M , Tonkin‐Crine S , Pruthi R , et al. Patient attitudes towards kidney transplant listing: qualitative findings from the ATTOM study. Nephrol Dial Transplant. 2014;29:2144‐2150. doi:10.1093/ndt/gfu188 24997006 PMC4209877

[7] Kittiskulnam P , Johansen KL . The obesity paradox: a further consideration in dialysis patients. Semin Dial. 2019;32:485‐489. doi:10.1111/sdi.12834 31338891 PMC6848753

[8] Chirban A , del Valle DD , Coe T , et al. Elements of weight management among pre‐kidney transplant candidates: the patient perspective. Transpl Int. 2024;37:12735. doi:10.3389/ti.2024.12735 38855426 PMC11160837

[9] Suresh A , Robinson L , Milliron BJ , et al. Approaches to obesity management in dialysis settings: renal dietitian perspectives. J Ren Nutr. 2020;30:561‐566. doi:10.1053/j.jrn.2020.01.021 32144072 PMC7483414

[10] Ryan L , Coyne R , Heary C , et al. Weight stigma experienced by patients with obesity in healthcare settings: a qualitative evidence synthesis. Obes Rev. 2023;24:e13606. doi:10.1111/obr.13606 37533183

[11] Harhay MN , Klassen AC , Gunen B , et al. Patient and health care professional perspectives on addressing obesity in ESKD. Am J Kidney Dis. 2023;82:419‐428. doi:10.1053/j.ajkd.2023.02.005 37086964 PMC10524159

[12] Chadban SJ , Ahn C , Axelrod DA , et al. KDIGO clinical practice guideline on the evaluation and management of candidates for kidney transplantation. Transplantation. 2020;104:S11‐S103. doi:10.1097/TP.0000000000003136 32301874

[13] Vanek L , Kurnikowski A , Krenn S , Mussnig S , Hecking M . Semaglutide in patients with kidney failure and obesity undergoing dialysis and wishing to be transplanted: a prospective, observational, open‐label study. Diabetes Obes Metab. 2024;26:5931‐5941. doi:10.1111/dom.15967 39375862

