# Supplementary material for: Burden of obesity in patients undergoing dialysis, and hopes associated with semaglutide treatment for transplant listing: An interview study
Source: Diabetes Obes Metab. 2025 Aug 25;27(11):6743–7. doi: 10.1111/dom.70011 (PMC12515747; doi:10.1111/dom.70011)
Supplement: Supplementary file 1 — Data S1. Supporting information. [file DOM-27-6743-s001.docx]

# **Supplementary Appendix**

## **Supplementary Methods**

### ***Supplementary Methods 1 - Interview guide:***

The following list represents the final, expanded version of the interview guide developed in accordance with the principles of grounded theory.

Kidney Disease:

How are you currently regarding your kidney disease? How did your health/condition evolve since you found out you had CKD? Which limitations arise thereof in your daily living?

Dialysis:

You are currently undergoing dialysis care. How do you cope with this treatment?

Kidney Transplantation:

Do you know if you are eligible for kidney transplantation? What are your thoughts on kidney

transplantation and its listing process?

Burden of Obesity:

- Has your body composition ever affected your health? (and how?) Have you ever experienced any restrictions due to your body composition?

- What body image ideal do you have? Which body image do you seek to achieve?

- Do you think your body composition sets you apart from other patients with CKD?

- Have you personally ever felt too heavy for any kind of activity or treatment?

- Did you ever have the notion, that doctors label you ill/sick only due to your overweight?

- How do you feel about not being eligible for all treatments due to your body composition?

(transplantation listing)

- Concerning your body composition – have you sought assistance from either medical professionals or otherwise to help you lose weight? What have you tried to lose weight?

Additional questions for patients with previous semaglutide obesity therapy:

- Could you tell me how you reacted when you first heard of the semaglutide treatment?

- Did you seek information from sources outside the medical setting?

- How did you tolerate the therapy at the beginning? Has something changed over the course of your treatment?

- How did the treatment with semaglutide affect your kidney health/body composition/other

comorbidities (e.g. diabetes)?

Additional questions on gender-related aspects:

- Do you think that men and women have different experiences with kidney disease or obesity?

- Whom do you think does obesity affect more in day-to-day life?

- Do you think that there is a difference between men and women and how they treat their kidney disease?

- Do you think men and women are treated differently in kidney care?

### ***Supplementary Methods 2:***

We conducted a qualitative interview study informed by grounded theory methodology to explore participants' experiences and perspectives on the impact of obesity in dialysis setting. Participants were purposively sampled to ensure a range of relevant experiences, and recruitment continued until thematic saturation was reached. The final sample consisted of 25 patients recruited from the Kuratorium for Dialysis and Transplantation e.V. Kidney Center Weiden in Germany (ten participants, of whom six had previous experience with semaglutide therapy for obesity treatment) and from the General Hospital Vienna (Austria) (15 participants). No fixed sample size or participant ranking was defined a priori; instead, recruitment continued until thematic saturation was achieved. Semi-structured interviews were used to allow for both consistency across participants and flexibility to follow emergent themes. The initial interview guide was informed by team discussions and literature review (Supplementary Table S1) and flexibly adapted throughout data collection and analysis in response to emerging themes according to the principles of grounded theory. Interviews were audio-recorded, transcribed verbatim, and analyzed using constant comparative methods. Coding was carried out iteratively, beginning with open coding to identify key concepts, followed by axial and selective coding to refine categories and develop a coherent theoretical framework grounded in the data. Reflexivity was maintained throughout the analysis to account for potential researcher bias. Artificial intelligence tools (ChatGPT-OpenAI, Endnote with AI integration-Clarivate, Elicit-Ought and Paperpal Preflight) supported the literature review, translation, and manuscript preparation, with all outputs reviewed for accuracy.

Table S1: References used to inform the development of the interview guide.

| 1 | Ryan, L. *et al.* Weight stigma experienced by patients with obesity in healthcare settings: A qualitative evidence synthesis. *Obes Rev* **24**, e13606 (2023). <https://doi.org:10.1111/obr.13606> |
| --- | --- |
| 2 | Tomiyama, A. J. *et al.* How and why weight stigma drives the obesity ‘epidemic’ and harms  health. *BMC Medicine* **16**, 123 (2018). <https://doi.org:10.1186/s12916-018-1116-5> |
| 3 | Pearl, R. L. *et al.* Association between weight bias internalization and metabolic syndrome among treatment-seeking individuals with obesity. *Obesity (Silver Spring)* **25**, 317-322 (2017). <https://doi.org:10.1002/oby.21716> |
| 4 | O'Shea, D., Kahan, S., Lennon, L. & Breen, C. Practical Approaches to Treating Obesity: Patient  and Healthcare Professional Perspectives. *Adv Ther* **38**, 4138-4150 (2021). <https://doi.org:10.1007/s12325-021-01748-0> |
| 5 | Tommel, J. *et al.* "What matters to you?": The relevance of patient priorities in dialysis care for assessment and clinical practice. *Semin Dial* **36**, 131-141 (2023). <https://doi.org:10.1111/sdi.13080> |
| 6 | Palmer, S. C. *et al.* Dietary and fluid restrictions in CKD: a thematic synthesis of patient views from qualitative studies. *Am J Kidney Dis* **65**, 559-573 (2015). <https://doi.org:10.1053/j.ajkd.2014.09.012> |
| 7 | Calestani, M. *et al.* Patient attitudes towards kidney transplant listing: qualitative findings from the ATTOM study. *Nephrol Dial Transplant* **29**, 2144-2150 (2014). <https://doi.org:10.1093/ndt/gfu188> |
| 8 | Suresh, A. *et al.* Approaches to Obesity Management in Dialysis Settings: Renal Dietitian Perspectives. *J Ren Nutr* **30**, 561-566 (2020). <https://doi.org:10.1053/j.jrn.2020.01.021> |
| 9 | Chirban, A. *et al.* Elements of Weight Management Among Pre-Kidney Transplant Candidates: The Patient Perspective. *Transpl Int* **37**, 12735 (2024). <https://doi.org:10.3389/ti.2024.12735> |
| 10 | Lopez-Vargas, P. A. *et al.* Patient awareness and beliefs about the risk factors and comorbidities associated with chronic kidney disease : A mixed-methods study. *Nephrology (Carlton)* **22**, 374-381 (2017). <https://doi.org:10.1111/nep.12829> |
| 11 | Vanek, L. *et al.* Patient and Caregiver Perspectives on Gender Disparity in Chronic Kidney Disease: Questionnaire Survey, Based on an Interview Study. *Am J Nephrol* **55**, 561-582 (2024). <https://doi.org:10.1159/000540850> |
| 12 | Lewandowski, M. J. *et al.* Patient and Caregiver Perspectives on Gender Disparity in CKD: An Interview Study. *Kidney360* **6**, 227-235 (2025). <https://doi.org:10.34067/KID.0000000594> |
| 13 | Rand, K. *et al.* "It is not the diet; it is the mental part we need help with." A multilevel analysis of psychological, emotional, and social well-being in obesity. *Int J Qual Stud Health Well-being* **12**, 1306421 (2017). <https://doi.org:10.1080/17482631.2017.1306421> |
| 14 | Harhay, M. N. *et al.* Patient and Health Care Professional Perspectives on Addressing Obesity in ESKD. *American Journal of Kidney Diseases* **82**, 419-428 (2023). <https://doi.org:10.1053/j.ajkd.2023.02.005> |
| 15 | Jimenez-Loaisa, A., Beltran-Carrillo, V. J., González-Cutre, D. & Jennings, G. Healthism and the experiences of social, healthcare and self-stigma of women with higher-weight. *Social Theory & Health* **18**, 410-424 (2020). |
| 16 | Meleo‐Erwin, Z. C. ‘No one is as invested in your continued good health as you should be:’an exploration of the post‐surgical relationships between weight‐loss surgery patients and their home bariatric clinics. *Sociology of health & illness* **41**, 285-302 (2019). |
| 17 | O’Donoghue, G. *et al.* A qualitative exploration of obesity bias and stigma in Irish healthcare; the patients’ voice. *PloS one* **16**, e0260075 (2021). |
| 18 | Paine, E. A. “Fat broken arm syndrome”: Negotiating risk, stigma, and weight bias in LGBTQ healthcare. *Social science & medicine* **270**, 113609 (2021). |
| 19 | Raves, D. M., Brewis, A., Trainer, S., Han, S.-Y. & Wutich, A. Bariatric surgery patients' perceptions of weight-related stigma in healthcare settings impair post-surgery dietary adherence. *Frontiers in psychology* **7**, 1497 (2016). |
| 20 | Tong, A. *et al.* Nephrologists' Perspectives on Gender Disparities in CKD and Dialysis. *Kidney Int Rep* **7**, 424-435 (2022). <https://doi.org:10.1016/j.ekir.2021.10.022> |
| 21 | Tong, A., Sainsbury, P. & Craig, J. C. Consolidated criteria for reporting qualitative research (COREQ): a 32-item checklist for interviews and focus groups. *International Journal for Quality in Health Care* **19**, 349–357 (2007). <https://doi.org:https://doi.org/10.1093/intqhc/mzm042> |

## **Supplementary Results**

### ***Supplementary Table S2:***

Table S2: Participant Demographic and Self-Reported Clinical Characteristics (N=25)

| **Characteristic** | | **N (%)** | **Characteristic** | | | **N (%)** |
| --- | --- | --- | --- | --- | --- | --- |
| Gender | |  | Relationship Status | | |  |
|  | Men | 14 (56%) |  | Married or living in partnership | | 14 (56%) |
|  | Women | 11 (44%) |  | Single | | 11 (44%) |
| Age category | |  | Employment status | | |  |
|  | 20s | 1 (4%) |  | Unemployed | | 11 (44%) |
|  | 30s | 1 (4%) |  | Employed | | 9 (36%) |
|  | 40s | 3 (12%) |  | Retired | | 5 (20%) |
|  | 50s | 7 (28%) | Other physical or psychiatric conditions | | |  |
|  | 60s | 9 (36%) |  | Cardiovascular disease | | 15 (60%) |
|  | 70s | 4 (16%) |  | Cancer | | 4 (16%) |
| Race | |  |  | Polyneuropathy | | 3 (12%) |
|  | White | 25 (100%) |  | Depression/anxiety | | 3 (12%) |
| Cause/type of Kidney disease | |  | Vintage years | | | Mean 3.75 years |
|  | Diabetes | 7 (28%) | History of Transplantation | | |  |
|  | IgA-nephropathy | 5 (20%) |  | Never undergone transplantation | | 21 (84%) |
|  | Hypertension | 5 (20%) |  | Status post one kidney transplantation | | 2 (8%) |
|  | Other/unknown | 8 (32%) |  | Status post more than one kidney transplantation | | 2 (8%) |
| Type of Dialysis | |  | Interview Language | | German | 25 (100%) |
|  | Thrice-weekly Hemodialysis | 24 (96%) | Interview Duration | | Mean | 22min |
|  | Daily Peritoneal dialysis | 1 (4%) |  | |  |  |

## **Supplementary Results 2: Key Areas of Patient Interest Beyond Obesity**

All participants raised the following three themes: diminishing quality of life, patient’s role within the healthcare system and gender-related considerations (Figure S1, Table S3). Although these topics were not directly linked to obesity by participants, their consistent mention across all interviews supported their inclusion in the supplementary findings for completeness.

### ***Theme 5 - Diminishing quality of life***

Participants felt that kidney failure and dialysis had taken away both their private and professional lives and had significantly deteriorated their quality of life. Prolonged dialysis vintage was particularly linked to health downturns. Patients reported handling these life changes with varying success, ranging from resentment over resignation to acceptance.

***Theme 6 - Patient’s role within the healthcare system***

Owing to their prolonged medical history, patients on dialysis were deeply familiar with the workings within the healthcare system and the interdependence between themselves and healthcare providers. Communication difficulties between patients and doctors, as well as the workload of healthcare professionals, were listed as factors that negatively affected patient-doctor relations.

### ***Theme 7 - Gender-related considerations***

Participants brought up gender differences by addressing gender stereotypes and commenting on the differences between men and women in relation to health and disease self-management. Gender stereotypes centered around the notion of women as the “weaker gender” and touched on the implications for transgender medicine. In terms of health self-management, women highlighted the importance of maintaining a positive outlook and not giving up. Not aggravating their health situation and preserving independence by relying more on their gut feelings and actively participating in decision-making was important to women, while men refrained from meddling in decisions, were more willing to relinquish control to doctors, and to let others take over their tasks.

Figure S1: Experiences of Patients with Obestiy Undergoing Dialysis – Key Areas of Patient Interest Beyond Obesity.

Table S3: Key Areas of Patient Interest Beyond Obesity – Themes, Subthemes and Supporting Quotes. w – woman patient, m – man patient.

| **THEME 5: DIMINISHING QUALITY OF LIFE** | | | |
| --- | --- | --- | --- |
|  | ***Restrictions in private and professional life*** | | |
|  |  | “You never know what kidney disease will take away from you this time - whether it's food, water or activities.” | w, 60s |
|  |  | “My quality of life has been reduced to mere survival.” | m, 60s |
|  |  | “How dialysis affects my private life? What kind of private life‽” | w, 40s |
|  |  | “Sometimes I have good days, sometimes bad ones. Occasionally, I have days where I can't do anything but sit around. Working just isn't in the picture then.” | m, 60s |
|  | ***Increasing burden with dialysis vintage*** | | |
|  |  | “Over the years, it has become increasingly exhausting. The body feels that it is being overstrained more and more during dialysis.” | m, 60s |
|  |  | “Dialysis days are completely ruined either way. … The body keeps giving way, and everything becomes more difficult.” | w, 60s |
|  | ***Coming to terms with kidney failure and dialysis*** | | |
|  |  | “Dialysis is a life changing event that you must learn to cope with. Some people manage it, but I don’t.” | w, 50s |
|  |  | “They told me back then that I would definitely need dialysis at some point. I was already mentally prepared because they had warned me. Then the time came, and I just had to accept it.” | m, 50s |
|  |  | “Dialysis is definitely a turning point in life. Three times a week. But I've been doing it for over three years now. You get used to everything.” | m, 60s |
|  |  | “I don't feel mentally burdened. It does take more than dialysis to throw me off course.” | m, 60s |
| **THEME 6: PATIENT’S ROLE WITHIN THE HEALTH CARE SYSTEM** | | | |
|  | ***Doctor as confidant*** | | |
|  |  | "I have never googled anything about my illness. I have never changed my doctor. That's where I receive my care, and no question ever remains unanswered. My second place of residence is my dialysis clinic." | m, 20s |
|  | ***Left at mercy of health care provider*** | | |
|  |  | “Doctors cannot heal; they can only provide the drugs. What does the devil's workshop offer, what do I take? As a patient, there's nothing else left to be done for me.” | m, 50s |
|  |  | “I would rather die than experience the ICU again. Inhumane. If someone works in the ICU, they should be psychologically evaluated to see if they can work there or if they'd rather work in a prison camp.” | w, 70s |
|  | ***Workload of healthcare professionals*** | | |
|  |  | "In the healthcare system, everyone is stretched too thin. There is too little staff everywhere. Everything must be done quickly. That's how mistakes happen.” | w, 60s |
|  |  | “Too many patients and too little staff. … It's not that the staff is bad. I can attest that everyone is doing their best. They're just overworked.” | m, 60s |
|  | ***Communication difficulties*** | | |
|  |  | “Every time, the doctors tell me something different. That's just not right.” | w, 70s |
| **THEME 7: GENDER-RELATED CONSIDERATIONS** | | | |
| **Gender stereotypes** | | | |
|  | ***Women as the “weaker gender”*** | | |
|  |  | “As a woman, you're quickly labeled hysterical.” | w, 30s |
|  |  | “As a woman, you must stand up for yourself even more. Unfortunately, that's the way it is. You must establish for yourself: this far and no further." | w, 70s |
|  |  | "I won't let myself be taken for a fool. While my male dialysis colleague discusses medical-scientific questions with the doctors, I get told that I wouldn't understand them anyway by the doctors … The assumption that man automatically equals expert." | w, 30s |
|  | ***Implications for transgender medicine*** | | |
|  |  | “Men doctors of various ages apparently aren’t taught at university that women patients exist. Now there's a discussion on how to handle transgender individuals. In the clinical routine, it hasn’t even sunk in that there are women. The care implications for transgender individuals will be ignored just like, apparently, for several hundred years of medical history, the existence of women has been ignored.” | w, 30s |
| **Health and disease self-management** | | | |
|  | ***Women with positive outlook*** | | |
|  |  | “You must never give up—never. You always have to keep on fighting.” | w, 60s |
|  |  | “Tick it off and move on. Sometimes it's better to be more of a realist than an optimist." | w, 50s |
|  |  | "I won't let kidney disease limit me. I wanted my life back and haven't let the disease determine my life. | w, 40s |
|  |  | “Letting yourself get discouraged gets you nowhere, because it doesn't change the situation. You always have to look for the silver lining.” | w, 60s |
|  | ***Women retaining control over their health*** | | |
|  |  | "Sometimes I can feel that I have a certain amount of weight. Based on that I decide when it's enough with the fluid removal.” | w, 40s |
|  |  | “A less intelligent person lets everything happen to them and doesn't stand up for themselves. You can't do that with me.” | w, 70s |
|  |  | “I wouldn’t rely on anyone else for scheduling appointments. I want to handle that myself.” | w, 60s |
|  |  | “It's better to go to the hospital one time too many than to be picked up from the floor at home. I prefer to be cautions, it's better to be safe than sorry.” | w, 50s |
|  | ***Men relinquishing control over their health*** | | |
|  |  | “I don't meddle with the determination of the dry weight.” | m, 60s |
|  |  | “I wouldn't force my wife to take over, but if she wanted to, I wouldn't be against it.” | m, 20s |
|  |  | “You can see significantly more men at hemodialysis sessions than women. I assume that many women opt for peritoneal dialysis. Therefore, I guess that men are just less independent than women.” | w, 60s |

## **Acknowledgments**

Author Contributions: L.V.: conceptualization, data curation, formal analysis, investigation, methodology, writing – original draft, and writing – review and editing. E.S.: conceptualization, data curation, formal analysis, investigation, and writing – review and editing. A.K., S.K., S.M., and J.N.: conceptualization, methodology, validation, and writing – review and editing. M.H.: funding acquisition, project administration, resources, supervision, conceptualization, validation, and writing – review and editing.

Conflict of Interest declaration: The authors declare that the research was conducted in the absence of any commercial or financial relationships that could be construed as a potential conflict of interest.

Funding Sources: This study was supported by Grant No. KL754-B from the Austrian Science Fund.

Data Access Statement: All data generated or analyzed during this study are included in this article and its supplementary material files. Further inquiries can be directed to the corresponding author.
